# Epithelioid Myofibroblastoma in an Old-Male Breast: A Case Report with MRI Findings

**DOI:** 10.1155/2015/934163

**Published:** 2015-07-30

**Authors:** Seyma Yildiz, Zuhal Gucin, Ezgi Basak Erdogan

**Affiliations:** ^1^Department of Radiology, Bezmialem Vakif University, Fatih, Istanbul 34093, Turkey; ^2^Department of Pathology, Bezmialem Vakif University, Fatih, Istanbul 34093, Turkey; ^3^Department of Nuclear Medicine, Bezmialem Vakif University, Fatih, Istanbul 34093, Turkey

## Abstract

Myofibroblastoma of the breast (MFB) is a very rare benign stromal tumor. In recent years, increase in mammographic screenings has resulted in increased diagnosis of MFB. Most cases are old males and postmenopausal women. MFB may be confused as malignant, clinically, morphologically, or by imaging. Immunohistochemistry is essential for final diagnosis in these cases. We report a case of a pathologically diagnosed MFB in an 80-year-old male patient who had coexisting prostate cancer and describe its imaging characteristics, especially magnetic resonance imaging (MRI). In this paper, histopathological and MRI findings of the MFB were discussed.

## 1. Introduction

Myofibroblastoma of the breast (MFB), which arises from myofibroblasts, was first defined in 1987 by Wargotz et al. [1]. MFB is an extremely rare benign stromal tumor, but with the increase in mammographic screenings it is being diagnosed more often. In the literature, most cases of MFB are males and women of 41–85. MFB can be found in any tissue in the body but mostly occurs in the breast. Its prevalence is less than 1% of all breast tumors [2].

Morphologically, wide variants of MFB have been described, including cellular, infiltrative, epithelioid, deciduoid, lipomatous, collagenised, and myxoid [2, 3].

Both clinically and in imaging this extraordinary tumor may be confused for malignancy. A few cases have been accompanied with gynecomastia or some other conditions such as chest wall trauma, irradiation for breast carcinoma, scar tissue at surgical incision sites, and synchrone or metachrone organ malignancies; most cases are sporadic [3].

Herein we report a case of a pathologically diagnosed MFB in an 80-year-old male patient who had coexisting prostate cancer and describe the imaging characteristics, especially magnetic resonance imaging (MRI).

## 2. Case Report

An 80-year-old male was admitted to our hospital after he found a painless mass in the upper inner quadrant of his left breast. The patient had no history of breast injury or systemic disease except hypertension. His physical examination disclosed a nontender, hard, mobile mass with regular contour in the upper inner quadrant of the left breast. Axillary lymph nodes were not palpable and the overlying skin showed no retraction.

**Figure 1:**
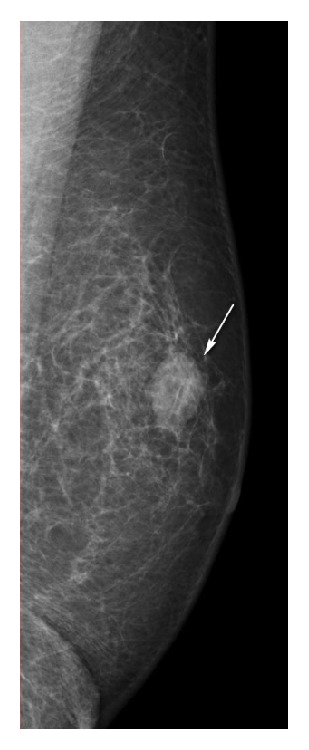
Myofibroblastoma of the breast of an 80-year-old man. A left, mediolateral oblique mammogram revealed a well-defined, moderate density mass (arrows) in the upper inner quadrant of the left breast.

The patient was initially evaluated by mammography (MG). The MG showed a 3.0 × 2.0 cm well-defined, round-shaped, medium-density mass in the upper portion of the left breast but no associated calcifications or architectural distortion (Figure 1) nor gynecomastia. After the MG evaluations, the lesion was evaluated by ultrasonography (US) examination, which showed a well-defined homogeneous hypoechoic solid mass of the left breast. No acoustic shadowing or increase through transmission was apparent.

**Figure 2:**
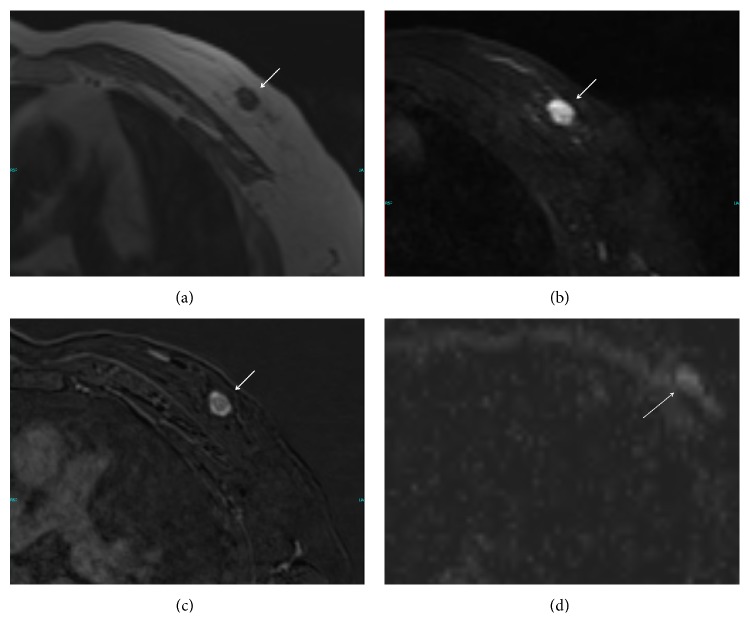
Magnetic resonance imaging findings of the breast mass. (a) T1-weighted spin echo MR image shows a focal mass with low signal intensity and smooth borders. (b) The fat-suppressed fast spin echo T2-weighted MR image shows a focal mass with high signal intensity. (c) Gd-enhanced fat-suppressed T1-weighted MR image shows strong heterogeneous enhancement of the mass and linear areas of low signal intensity in keeping with internal septations. (d) ADC value of the mass.

The patient next underwent bilateral dynamic contrast enhanced magnetic resonance imaging (DCE-MRI) and diffusion weighted imaging (DWI) via a 1.5 Tesla MR. Kinetic and morphologic analyses were performed on DCE-MRI. The lesion was oval, with a circumscribed margin. The signal intensity of the mass showed hypointense on precontrast T1-weighted MR (Figure 2(a)) and hyperintense on T2-weighted images (Figure 2(b)). Gd-enhanced fat-suppressed T1-weighted MR images showed early strong enhancement of the mass with nonenhancing internal septations (Figure 2(c)). Apparent diffusion coefficient (ADC) maps were used for ADC measurements. The lesion ADC value was 2.280 × 10^−3^ mm^2^/s (Figure 2(d)).

**Figure 3:**
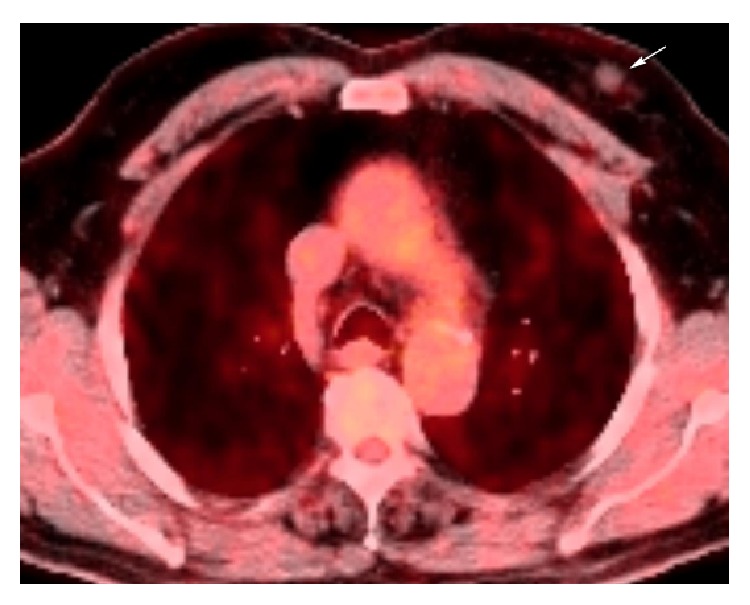
Axial 18-fluorine deoxyglucose positron emission tomography (18F-FDG-PET)/computed tomography (CT) fusion images and PET/CT fusion sections showing focal minimal FDG uptake in left breast.

For staging of malignant lesions, F18-fluoro-2-deoxyglucose positron emission tomography (FDG/PET) was performed to the patient, who had been prediagnosed with breast cancer. In the upper-left inner quadrant of his breast a round-shaped, nodular lesion was detected with a 12 mm diameter and a hypodense center with low FDG uptake (SUV max: 1.2) (Figure 3).

US-guided core needle biopsy of the mass from the breast was performed for histologic examination and exact diagnosis. The pathological results of the core biopsy were inadequate for differentiating between invasive carcinoma and a benign lesion. Therefore surgical excision was performed.

**Figure 4:**
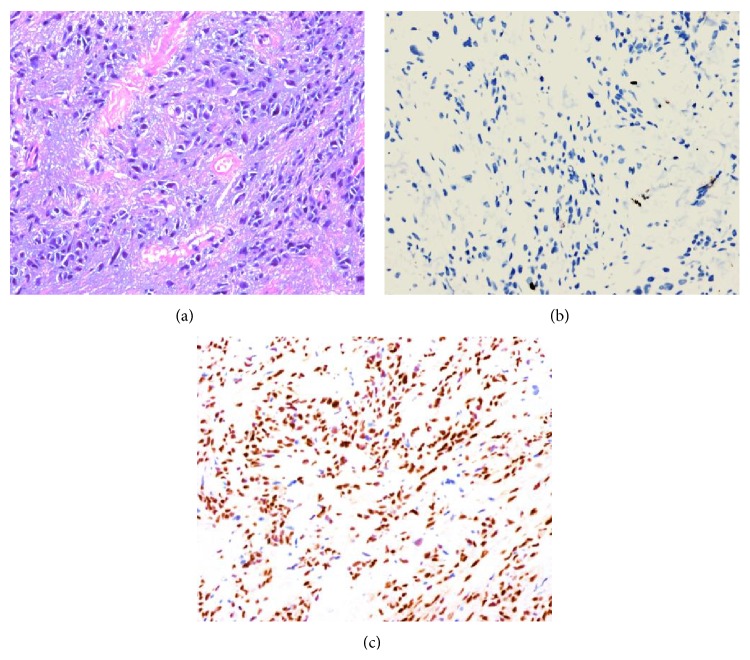
Pathologic findings of the breast lesions. (a) Overall appearance: tumor cells arranged in single file reminiscent of invasive lobular carcinoma (H&E ×200). (b) Negative for pankeratin (IHC ×100). (c) Estrogen receptor positivity (IHC ×100).

The lesion was composed of a well-circumscribed but not encapsulated proliferation of round, epithelioid-shaped myofibroblastic cells configured in single file or as small clusters in a vascularized, weakly collagenized stroma. Its histologic appearance resembled an invasive lobular carcinoma (Figure 4(a)). Immunohistochemistry revealed that the tumor cells were negative for keratin (Figure 4(b)) but positive for antismooth muscle actin and estrogen receptor (Figure 4(c)). These findings are characteristic of epithelioid myofibroblastoma.

## 3. Discussion

An MFB of the breast is an exceedingly rare tumor which is composed of myofibroblasts, and it may be confused clinically and in imaging for malignancy. This tumor was first defined in 1987 by Wargotz et al. [1]. In recent years, increase in mammographic screenings has resulted in increased diagnosis of MFB. Although MFB can be found in a wide age-range of patients from 1 to 87 years [3–6], most cases are postmenopausal women [7].

In the literature, few cases of MFB were accompanied with gynecomastia or some other conditions such as chest wall trauma, irradiation for breast carcinoma, scar tissue at surgical incision sites, and synchrone or metachrone organ malignancies; most cases are sporadic [5]. This extraordinary tumor causes some potential diagnostic pitfalls, not only because of its rarity, but also because of its morphological diversity. Histologic features of typical forms are composed of innocuous spindle cells. Atypical cells may be seen, especially in cellular, epithelioid, myxoid, and deciduoid variants, which actually represent degenerative features [3]. Differential diagnosis may be difficult in core biopsies, especially with unusual variants. Invasive lobular, apocrine, and metaplastic carcinomas are main potential confounding conditions [3]. Immunohistochemistry is essential for final diagnosis in these cases.

In the literature, cases of breast malignancies concomitant with MFB have been reported, but in males prostate adenocarcinoma coexisting with MFB has not. In our case, prostate adenocarcinoma detected in the course of further investigation of breast mass was considered coincidental and not psychopathologically related with the MFB.

There is not enough knowledge about radiologic finding especially DCE-MRI finding of male breast MFB in the literature [8, 9]. DCE-MRI is an important imaging modality and is increasingly used to detect and characterize breast lesions. Moreover, on DWI imaging, malignant lesions have low values of ADC whereas benign lesions have high values of ADC [10]. Some myofibroblastic tumor cases reported in the literature were evaluated with DCE-MRI [8, 9], but we did not find any information using the DWI. In our case, the lesion had significantly higher ADC values as seen in benign lesions (the lesion ADC value was 2.280 × 10^−3 ^mm^2^/s) in ADC map. In cases that MFB misdiagnosed as breast malignancies, ADC values may be helpful in differentiating MFB from malignant lesions. To the best of our knowledge, this is the first case of MFB studied with MR diffusion findings of lesions in a male breast.

## 4. Conclusion

An MFB of the breast is an extremely rare stromal benign tumor. The diagnosis of MFB may sometimes be difficult and misdiagnosed as malignancy leading to unnecessary interventional procedures of breast. Clinicians should pay attention to all clinical features, radiologic findings, and pathological results including immunohistochemistry. DW-MR imaging may be helpful to differentiate MFB from malignant breast tumors.
